# Perspectives of Obstetricians and Women with a History of Prior Cesarean Birth Regarding Subsequent Mode of Birth in Trifinio and Coatepeque, Guatemala

**DOI:** 10.26502/ogr074

**Published:** 2022-01-21

**Authors:** Andrea Jimenez-Zambrano, Kathryn Feller, Claudia Rivera, Angela Marchin, Antonio Guillermo Bolanos, Edwin Asturias, Hector Rodas, Margo S Harrison

**Affiliations:** 1 University of Colorado, Department of Pediatrics, CO, USA; 2 Colorado School of Public Health, Center for Global Health, CO, USA; 3 University of Colorado Department of Obstetrics and Gynecology, CO, USA; 4 Center for Human Development, Ritalhuleu, Guatemala; 5 Department of Obstetrics and Gynecology, Coatepeque Hospital, Coatepeque, Guatemala

**Keywords:** Beliefs, Attitudes, Practices, Mode of Birth After Cesarean Birth

## Abstract

**Background:**

The decision regarding delivery in the context of a prior cesarean birth is complex because both trial of labor after cesarean and elective repeat cesarean birth have risks and benefits.

**Purpose:**

Our research objective was to understand the perspective of women and obstetricians regarding factors influencing mode of birth for women with a history of prior cesarean.

**Methods:**

In February 2020, qualitative data was collected at Coatepeque Hospital in Coatepeque, Guatemala. In-depth interviews were conducted with obstetricians and women at the Center for Human Development in the Southwest Trifinio region. Interviews were recorded, transcribed, translated, and analyzed using conceptual content analysis of key informant interviews to analyze the meaning of themes and concepts related to mode of delivery for women with a history of prior cesarean birth.

**Results:**

Women described feeling conflicted about their preferences on the location and attendant of their future births, but suggested that the hospital setting, and physician providers were more capable of managing complications. Physicians felt trial of labor after cesarean was the safer option but described multiple reasons that made repeat cesarean birth the more common mode of birth.

**Conclusions:**

There is a need for innovative approaches to patient messaging and education around mode of delivery after a prior cesarean in the Southwest Region in Guatemala. Findings from this study underscore the need to improve the quality and dissemination of the educational information given, medical history collected during prenatal care, and pain control during labor. Finally, there is a need for obstetric training to support vaginal birth in the facility setting for the successful implementation of evidence-based practices around trial of labor after cesarean at Coatepeque Hospital.

## 1. Introduction

Cesarean birth rates are increasing in Guatemala and in the Latin American region [1]. In 2013 the cesarean birth rate in Guatemala was around 16%, in 2015 it was around 26%, and in our prior research we found that in the Southwest Trifinio, cesarean birth rates increased from 30% in 2015 to 45% in 2017 [2–4]. As the population of women who undergo cesarean birth increases, so does the population of women with a history of prior cesarean birth. Once a woman has a scarred uterus from a cesarean, she can deliver by elective repeat cesarean or attempt a trial of labor (vaginal birth) after cesarean [5]. For properly selected women, trial of labor after cesarean is a safe, evidence-based choice; it is estimated that 60 – 80% of women would achieve vaginal birth if they tried to do so [5]. However, outcomes of trial of labor can be catastrophic if mismanaged [5, 6]. Currently, women with a history of prior cesarean who choose elective repeat cesarean birth account for the largest proportion of the overall cesarean birth rate in Guatemala [7, 8]. Prior research from varied global settings has suggested that characteristics associated with elective repeat cesarean birth include increased maternal age and education, information received after the prior cesarean birth, participation in antenatal courses, that women perceive a suboptimal quality of care for vaginal birth, facilities are understaffed without standard protocols, and there is a lack of privacy and dignity [9, 10]. Our prior research from our community in Guatemala found that characteristics associated with repeat cesarean birth were reduced parity, delivering at a facility (as compared to home), and delivered by a physician; the latter two characteristics were highly correlated as expected [11].

Our research objective was to understand the perspective of women with a history of prior cesarean birth regarding their desires for future births, as well as the perspective of the obstetricians in the facility (Coatepeque Hospital) where they commonly deliver. Coatepeque is a public referral facility that provides labor and delivery care to all admitted patients free of charge. We intended for this study to provide context for any future interventions that might be developed in this region regarding mode of birth after a prior cesarean birth.

## 2. Methods

Physicians were approached and recruited at their site of work at the Coatepeque Hospital in Coatepeque as they were engaged in clinical care and asked to participate in our study. If they wished to participate, they were brought the conference room, consented, and interviewed privately by the study team. The study was approved by the Colorado Multiple Institutional Review Board (COMIRB #19-0615), INCAP (CIE-REV 088/2019), and the Guatemalan Ministry of Health (#50-2019). Interviews with women took place in a consultation room at the Center for Human Development in the Southwest Trifinio region of Guatemala, an area at the intersection of three Guatemalan departments that borders Mexico. The clinic also houses community outreach programs that provide maternal and child health to pregnant women and children in the surrounding area, from which our study population was sampled. The study coordinator, the nursing supervisor of the maternal health program, recruited a sample of women who recently delivered by cesarean birth. To obtain the convenience sample, starting in November 2019, nurses who visited women for their postpartum visits offered participation in the study.

### 2.1 Data collection and analysis

**Figure 1:**
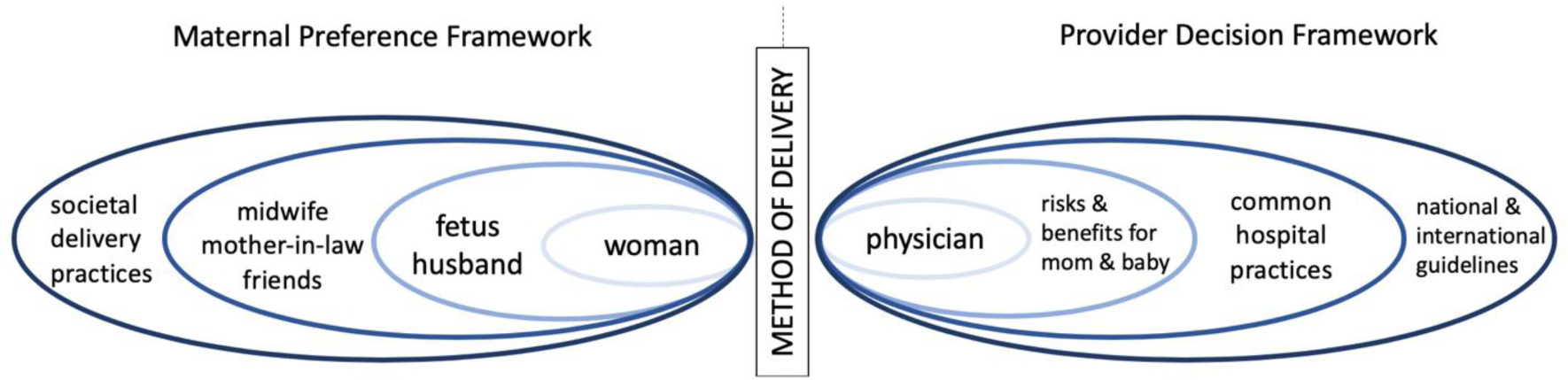
Socioecological Framework used to Develop Interview Guides.

Data collection took place from February 11 – 19, 2020, included semi-structured in-depth interviews, and all the data collection took place in Spanish with a native speaker. A Native-Speaker interviewer led all the physician interviews as well as the women interviews. Interviews were conducted with physicians (n=10) and with women who had a cesarean birth (n=20). The socioecological model (Figure 1) was used to guide our interview guides, but by the time the data was collected, the World Health Organization (WHO) had produced its own, similar socioecological framework for factors contributing to the use of cesarean birth at the local level [12]. The framework divides factors into those that contribute at the organizational and system level, the health professional level, women and community level, and medical level (Robson classification system for cesarean birth) [12]. Prior to analysis, we divided our codebook into these socioecological levels. The interview guides for the obstetricians focused on knowledge, attitudes, and practices related to mode of delivery for women with a history of prior cesarean birth at Coatepeque Hospital including clinical indications and social considerations based on a socioecological framework we developed in designing our study (Figure 1). The interview guides for women focused on their ideal future birth and their attitudes and beliefs about mode of delivery after a prior cesarean birth using the same framework. The interview guides were not adapted over the course of the study. All interviews were audio recorded and lasted between 15 – 45 minutes. A member of the research team also took detailed notes.

Accordingly, while we used an inductive approach to develop our codes, we used the WHO framework deductively to analyze our data. Data were analyzed using conceptual content. Using an inductive approach, a set of codes was developed from multiple readings of and immersion in the transcripts. All discrepancies in the code definitions and applications were reconciled through consensus. Codes were clustered into related categories which guided theme development. These themes were oriented to describe what the women understand and how they feel about method of delivery after cesarean, as well as the knowledge, attitudes, and practices of providers. Audio recordings were transcribed verbatim by a HIPAA-certified professional transcriptionist in the language of the interview (Spanish). Spanish-language interview transcripts were professionally translated into English. When the data were prepared, it was sent securely to the senior professional research assistant who stored the data on password protected servers. Translated transcripts were reviewed for integrity and uploaded into ATLAS.ti software in preparation for analysis in a de-identified format, with interviews saved as a combination of numbers and letters, allowing for anonymization of the content. The codebook was then applied to all transcripts by these members of the research team.

## 3. Results

Qualitative analysis of both the physicians and women’s interviews indicated key themes that emerged from the data: (1) System Factors, (2) Health Professional Factors, (3) Women Factors, and (4) Medical Factors that influence subsequent mode of birth after a cesarean [12]. System factors refers to professional power relationships, quality improvement, strength of the multidisciplinary team, commitment to use of evidence-based medicine, role of the hospital, financing structures, and the culture of intervention [12]. Health Professional Factors refers to provider beliefs about birth, their education and training, their beliefs about the need to reduce cesarean use, their beliefs about vaginal birth after cesarean and the doctor-patient relationship, their beliefs about women, the fear of blame, financial rewards associated with cesarean, and the convenience of cesarean birth [12]. Women Factors include women’s receptiveness to learning new information about birth, having multiple information sources, their previous birth experience, their choices and uncertainty about what will happen during labor and delivery, their fear and anxiety, and their emotional support systems [12]. Medical Factors refers to the women’s risk factors for cesarean birth based on their medical and obstetric characteristics [12].

### 3.1 System factors

Women described several factors that have important implications when making their decisions in relationship to system factors. They reported that anesthesia was only available with cesarean birth and that the hospital has more resources to manage birth complications than those available in the home setting. One woman shared why she delivered in the hospital by saying, “because it was the first time and first-time mothers cannot give birth at home because if you can’t have a normal birth, they can do a cesarean.” Another woman noted, “the doctor will do everything he can to prevent complications.” With respect to management of pain during labor, one woman explained, “I got scared and I told my husband that it was better if they did the cesarean because I couldn’t take the pain anymore.” Similarly, another participant described that “you suffer more having it normal and having a cesarean you’re only in pain for a little while.”

Women reported they make their decision related to mode of delivery after cesarean based on system resource factors, due to viewing the hospital in terms of a setting where they can have pain and complications managed, with little mention of economic factors. Physicians had more insight into the hospital system resources, which influenced the mode of delivery among women with a history of cesarean birth. A theme that repeatedly emerged was a lack of resou-rces in the hospital setting to manage the demand for cesarean birth. One physician reported, “It is deficient, we don’t have many stretchers, there is no surgical area for any complications, we do not have enough space,” and another said “we are a little short because we only have one obstetric room…we attend it 24 hours…however, we may meet with an elective and an urgent [cesarean],” while a final provider reported, “if we offered that [cesarean] to all the public, we wouldn’t be able to solve all of them.” They believed that “the problem is space, we only have one surgery room and anesthesiologist…it would be good to have more surgery rooms and more staff.”

Conversely, when asked if the hospital has resources to attend every woman with a history of prior cesarean to pursue a trial of labor, responses included, “yes, we are currently well prepared and have the resources required,” as well as, “yes, we have enough [resources].” When questioned about the economics of mode of delivery, physician responses included, “if a patient comes for a normal delivery her stay will be shorter, the family will spend less money on going or coming to see her…I think it will favor the cost of the patient,” and “yes, I mean, a vaginal delivery is theoretically cheaper than a cesarean delivery.” Physicians reported that the system resources seemed to favor vaginal birth after cesarean over repeat cesarean but given the drive toward repeat cesarean birth the hospital may be under-resourced.

### 3.2 Health professional factors

Physicians shared that mode of delivery was the woman’s choice, and that women and families often present having already made that choice. Regarding elective repeat cesarean birth, one physician reported, “for example, she comes and the patient says ‘I have a cesarean, I have no indication of having another cesarean, but I want a cesarean, I don’t want a normal delivery’.” A different provider went on to say, “it depends on the patient, I think its individual, it also depends on what the patient wants because she has the right to control what is done to her body.” Another doctor reported, “I explain something to her, but the mother-in-law explains otherwise, the neighbor explains something else, the husband explains something else. One explains, and then, not anymore, because the mother-in-law decided otherwise.” The doctors also described the influence of social media on the mode of birth decision, with one physician noting, “and they have seen all this from Facebook, from social networks and they publish everything.”

When probed specifically about counseling related to mode of delivery one doctor noted, “the soul of the population saw that everyone wants deliveries now by cesarean because they do not take time, they want to leave quickly…everything is shortened, without pain…our culture has made cesareans for the entire population the wrong option.” Another offered, “you give information about the benefits and risks, nevertheless, the majority of these patients already come up with the idea that it has to be cesarean because a relative told them.” Another theme noted was the difficulty of counseling on the complex topic of mode of birth given time and educational constraints. One physician described, “it depends on her education; apart from prenatal care, I think it depends on how much the patient can understand.” A colleague said, “one often encounters a language barrier…perhaps the terminology is too technical, and they don’t understand what we are telling them. One tries to speak to them in layman’s terms.”

Physicians clearly focus on post-operative counseling of women related to future fertility. However, the theme that emerged did not center around mode of future delivery, but rather the importance of pregnancy spacing and postpartum contraception. One physician explained, “here we have a doctor, family planning specialist, spends every day explaining to use a control method, to go with a method… because of the cesarean,” while another noted “the educational plan of discharge…this includes everything that is contraception…it would be preferable if we can space a little beyond one, two years minimum, or perhaps a little more time.” The physicians were very focused on preventing short interval pregnancy after cesarean birth.

When we asked about training in obstetric skills that might support a safe vaginal birth after cesarean such as external cephalic version or operative vaginal delivery (forceps/vacuum), the interviewees reported that they were not trained in these options, with some of the older providers reporting trainings but no current practice of the skills. With respect to operative vaginal birth they clarified, “no we don’t use that type of instrument due to fetal trauma,” and “they are rare. They are very rare,” and “we received the training many years ago, but what is not practiced is also forgotten.” Concerning external cephalic version training one doctor responded, “no, not for that exactly, no. We’d like to learn, yes, I mean if they’d teach us, then yes.” Health professional factors that seem to contribute to mode of birth after cesarean from the physician perspective include counseling on pregnancy spacing, difficulty with counseling patients because of their predilection toward cesarean birth, and lack of obstetric skills to provide alternatives to cesarean birth.

### 3.3 Women factors

When talking to participants regarding factors that influenced the mode of delivery, women were conflicted when describing their preference between the hospital vs. the home setting with a tradition birth attendant in terms of their plans for their next delivery. One woman said of providers, “I feel that a traditional attendant…I know she was trained, but it’s not the same as a doctor. Sometimes a doctor has not only studied, but they have the experience in knowing how to deliver children.” Conversely, another woman described, “I would want my next birth to be at home and for the traditional attendant to tend to me, and that my family would be there with me, supporting us.” Women do appreciate the risk associated with giving birth, regardless of the specific circumstances with one woman declaring, “When you are delivering a baby you can live or die, as they say, because it’s a great risk, but thank God everything was fine.”

Regarding the different modes of delivery, one woman noted, “A cesarean…I think it’s the easiest, the cesarean does hurt at first, but I feel the cesarean is safer than normal delivery.” Another woman had the opposite opinion, stating, “I think normal delivery is safer; you have the baby and practically that’s it.” This sentiment was echoed in another interview where a woman explained the counseling she received and her conclusion in response: “He tells me that it is better to have another cesarean. Some doctors think that a cesarean is better, others say that it’s better to have a normal delivery. I think a normal delivery is better.” Another woman, when asked about mode of delivery, responded, “well, if God allows me to have a normal delivery, I would like that.” Patients seem to believe that the hospital setting, and physician birth attendants are more capable of managing maternal and perinatal complications, but in the end, they may have alternate preferences based on their prior birth experience. Regarding women-related factors that were associated with mode of delivery among women with a history of prior cesarean from the physicians’ perspective, the decision to pursue repeat cesarean birth seems to be driven by the woman prefences with the doctors feeling they have little ability to influence that decision because they are limited by time and the health literacy of the patient.

### 3.4 Medical factors

Beyond a focus on a maximum of three cesarean births, women did not report nuanced conversations occurring about future fertility and mode of birth with respect to medical risk, with one woman exclaiming, “no, they didn’t tell me that, why am I going to lie?” When asked if she would like more information about the risks and benefits of future modes of birth, another woman said, “yes.. I would like someone who knows to explain…a nurse or a doctor, but a doctor would be better, and the information to my husband, too.” Many women reported that they were required to wait between two and five years after a cesarean birth to become pregnant again, per their providers. One respondent noted, “they told me it was risky for me to have a child in such a short time, that after my first child, I had to wait a long time before having a second one…due to the scar the cesarean could be very difficult.” Women’s understanding of medical factors related to mode of delivery after a prior cesarean was that many felt repeat cesarean was their only option, that it was essential to space pregnancies properly, and that the max number of potential cesareans was three.

Physicians felt the best mode of delivery was trial of labor after cesarean for women who qualified, “for the patient’s well-being…having a normal delivery is of lower risk.” However, the list of exclusion criteria for trial of labor was extensive; any maternal or fetal issue at presentation seemed to disqualify a woman from an attempt, with the most prominent concern being the interpregnancy interval. One physician stated, “it is a safe option if they meet the requirements, but most of the time patients have only one visit, they have a short interpregnancy interval, and that leads to making the decision.” In addition to risk factors and time elapsed from the last delivery, physicians were very concerned about proper prenatal care, with one obstetrician asserting, “she hasn’t done any checkups and she only comes at the time so the risks are higher and we don’t know anything about the patient.”

When doctors were asked about specific protocols used in the facility to manage women who opt for trial of labor after cesarean, one provider affirmed that, “we usually determine the approximate [fetal] weight through ultrasound…we also assess her pelvis…and we are constantly monitoring her during labor and delivery to see how she is doing. If at some point we notice that it is not working, we immediately stop it and we don’t give her any more time.” As stated previously, though, this option is reserved for women without any notable obstetric concerns, with one doctor declaring, “but a patient with a short time between pregnancies, an abnormal presentation or some risk factor, meconium, tachycardia; we cannot do vaginal delivery.” Another physician stated, “if they have an STD, they won’t get it either, like genital warts, we don’t offer it, they’re immediately scheduled for cesarean.” Trial of labor after cesarean was reportedly not an option for women without prenatal care, those with an unknown obstetric and medical history, those with any concerns or complications on admission, and those without proper spacing between pregnancies.

## 4. Discussion

### 4.1 Integration

Our main findings from this qualitative study were that the women interviewed were conflicted about their preferences on the location and attendant of their future births, but they described that the hospital setting, and physician providers were more capable of managing complications. Their primary concern was their infant’s well-being with consideration of their husbands’ opinions, their concerns about the pain of childbirth, and risks and benefits of cesarean versus vaginal birth for the intrapartum and postpartum courses. Women were very clear on the concepts that a maximum of three cesarean births was permissible and pregnancies must be spaced at least two years for the safety of their future pregnancies and deliveries. With respect to our aims of understanding the knowledge, attitudes, and practices of physicians practicing at Coatepeque Hospital related to mode of birth for women with a history of prior cesarean, physicians identified trial of labor after cesarean was the safer option, but that for many reasons repeat cesarean birth was the more common mode of birth. They cited patient’s preference, medical concerns related to not knowing patients’ medical histories well, as well as patient health literacy constraints as reason for high rates of repeat cesarean birth. They report that there is a culture accepting of cesarean birth both in the hospital and community settings, and that the hospital is being taxed with respect to time and resources to meet that demand, which reflects what prior literature has shown for Latin America [13]. They also identified that the lack of skilled prenatal care did not allow for a good assessment of risk factors, and that any obstetric concern or lack of proper pregnancy spacing precluded women from being permitted to pursue trial of labor after cesarean. Physicians admitted to not having been trained in external cephalic version or operative vaginal birth as a means of supporting vaginal birth after cesarean.

### 4.2 Limitations

Our study was limited by the convenience sampling of our populations both in the hospital and in the community setting. Though the lead interviewers were a native and a fluent Spanish speaker, their status as Americans may have influenced the responses of women in the Trifinio as well as physicians in the hospital.

## 5. Conclusions

Additional prenatal education around mode of birth after a prior cesarean are needed to narrow the gap between women’s preference for cesarean based on their personal experiences versus what is medically available for them. Findings from this study suggest that for physicians the incentive for repeat cesarean birth is compelled largely by patient and there is a culture of cesarean birth that is driving practice, rather than the resources to properly counsel patients and support trial of labor after cesarean. These findings serve as the basis for our current research to develop an innovative approach to patient messaging and education around mode of delivery after a prior cesarean. This approach will improve the quality and dissemination of information given and history collected during prenatal care, to pain control and obstetric training to support vaginal birth in the facility setting, and to the implementation of evidence-based practice around trial of labor after cesarean at Coatepeque Hospital.
